# Leaf-Fruit Trait Decoupling Along Environmental Gradients in Tropical Cryptocaryeae (Lauraceae)

**DOI:** 10.3390/plants15010126

**Published:** 2026-01-01

**Authors:** Wendi Zhao, Lifang Wang, Yu Song, Honglei Jiang, Xiali Guo

**Affiliations:** 1 Guangxi Key Laboratory of Forest Ecology and Conservation, State Key Laboratory for Conservation and Utilization of Subtropical Agro-Bioresources, College of Forestry, Guangxi University, Nanning 530004, China; 2 Laibin Jinxiu Dayaoshan Forest Ecosystem Observation and Research Station of Guangxi, Laibin 545700, China; 3 Key Laboratory of Ecology of Rare and Endangered Species and Environmental Protection (Ministry of Education) & Guangxi Key Laboratory of Landscape Resources Conservation and Sustainable Utilization in Lijiang River Basin, Guangxi Normal University, Guilin 541004, China; 4 Guangxi Laboratory on the Study of Coral Reefs in the South China Sea, Coral Reef Research Center of China, School of Marine Sciences, Guangxi University, Nanning 530004, China

**Keywords:** Lauraceae, functional traits, phenotypic plasticity, environmental gradients, morphological variation, tropical ecology, trait correlations

## Abstract

Cryptocaryeae, as a significant tribe within the Lauraceae family with important economic and ecological value, comprises over 850 species. Its common ancestor dates back to approximately 123 million years ago, in the early Cretaceous, originating in tropical Africa and Asia. Understanding how leaf and fruit functional traits of Cryptocaryeae trees (Lauraceae) respond to environmental fluctuations is crucial for protecting the structure and function of forest ecosystems. In this study, we investigated the influence of environmental factors on leaf and fruit morphological traits in the tropical tribe Cryptocaryeae. Based on an established phylogenetic framework for Cryptocaryeae, we compiled a dataset containing 17,117 morphological observations across 369 species. The analyzed traits included leaf length, leaf width, leaf area, fruit length, fruit diameter, and fruit size. Through analyzing trends of leaves and fruits morphological traits across the latitude and longitude and their relationship with environmental factors, and by quantifying the relative contributions of environmental factors to these traits, we demonstrated that leaf morphology exhibited distinct latitudinal and longitudinal zonation and was sensitive to environmental fluctuations, especially to temperature changes. In contrast, the change of fruit morphological traits was comparatively conservative in their variation, mainly affected by precipitation. These findings suggest that different plant traits may employ different trade-off strategies during environmental adaptation. Highlighting the importance of integrating ecological and evolutionary perspectives on leaf and fruit morphological traits of tropical Cryptocaryeae trees could provide insights into understanding plant environmental adaptation.

## 1. Introduction

As sessile organisms, plants develop corresponding survival strategies through continuous evolution to maximize adaptation to their environment [1]. Trait variation plays a crucial role in species evolution and the maintenance of biodiversity [2]. However, since the industrial revolution, global climate change and intensified human activities have profoundly altered environment conditions, increasing temperature and drought events now pose serious threats to plant growth and survival [3,4]. Consequently, many plant species are migrating northward to seek suitable habitat [5]. Studies indicate, however, that this northward migration occurs at a slower rate than climate change, primarily due to long generation times and landscape fragmentation [6,7]. Therefore, investigating the patterns of plant responses to climate change can help promote the conservation of forest ecosystems and maintain the stability of their structure and function.

Plants adaptation results from plant-environment interactions, occurring primarily through two mechanisms: genetic variation or phenotypic plasticity [8,9]. Phenotypic plasticity enables a faster response to variable or detrimental environmental conditions compared to genetic adaptation [8,10], allowing plants to flexibly cope with environmental changes [11]. This plasticity is prevalent in plants and forms the basis for optimal adaptation under fluctuating or transiently adverse conditions [12,13]. Through phenotypic plasticity, plants adjust their morphology, physiology, and reproduction strategies to enhance adaptability and optimize resource use across diverse environments [14,15]. However, maintaining high phenotypic plasticity imposes metabolic costs, as plants divert energy from growth and defense to sustain responsiveness to environmental change [16]. Therefore, understanding the role of phenotypic plasticity in regulating plant responses to environment stressors is crucial. Plant traits are specific manifestations of plant adaptation to environmental conditions serving as a bridge between the environment, individual plants, and broader ecological processes and functions [17]. Their intraspecific variation and phenotypic plasticity can help predict species distribution under global climate change [15]. Functional trait variability provides plants with adaptive strategies to cope with environmental shifts [14], and certain traits reflect core aspects of plant ecological strategies, which may be linked to phenotypic plasticity [18]. For example, leaves—vital photosynthetic organ—are key traits for resource acquisition [19], and variations in their morphology critically influence plant structure, function, and environmental adaptation [20,21]. Similarly, fruit traits significantly impact plant reproduction and population establishment [22], while seed size fundamentally determines seed dispersal effectiveness [23].

The biogeographic histories of numerous lineages within the Lauraceae family remain poorly understood, largely due to challenges in definitively assigning macrofossils to extant genera, generally pollen preservation, and the absence of sufficiently resolved or well-supported phylogenies. To address these issues, Song et al. employed plastome sequencing to reinvestigate the phylogenetic and biogeographic history of the tribe Cryptocaryeae. The present study builds directly upon the phylogenetic framework established by their findings to conduct subsequent related research. Cryptocaryeae, a significant tribe within the Lauraceae family, comprises over 850 species primarily. Its reproductive strategy relies heavily on vertebrates for seed dispersal, having coevolved with the phenology of multiple animal species. Molecular phylogenetic studies confirm Cryptocaryeae as a monophyletic group, including five subgroups: Aspidostemon, Beilschmiedia, Cryptocarya, Dahlgrenodendron and Eusideroxylon. Their common ancestor can be traced back to about 123 million years ago in the early Cretaceous, originating in tropical Africa or Asia. Distributed primarily across tropical regions globally (notably Asia, Australia, and South America), they are ecologically significant components of evergreen broad-leaved forests, many of which possess substantial ecological and economic value [24,25]. However, current research on Cryptocaryeae, predominantly focuses on phylogenetics—reconstructing phylogenetic trees using genomic methods [24,25,26,27]—with relatively fewer studies addressing the relationship between environmental adaptation and key morphological traits in this group, it may exhibit limited plasticity in these functional traits. In this study, we compile global functional trait data (leaf and fruit) for Cryptocaryeae species to investigate the role of phenotypic plasticity in regulating their environmental adaptation strategies. Our specific objectives are to: (i) test the hypothesis that leaf traits exhibit stronger spatial and environmental correlations than fruit traits, reflecting divergent phenotypic plasticity; (ii) quantify the relative contributions of climatic factors in shaping trait variation, thereby assessing the potential role of phenotypic plasticity; in Cryptocaryeae. We hypothesized that (1) leaf morphological traits are more likely to be significantly affected by latitude and longitude gradients and climatic factors than fruit morphological traits; (2) leaf morphological traits and fruit morphological traits may have different response patterns to environmental gradients and factors.

## 2. Results

### 2.1. Global Patterns of Species Distribution of Cryptocaryeae Trees

**Figure 4:**
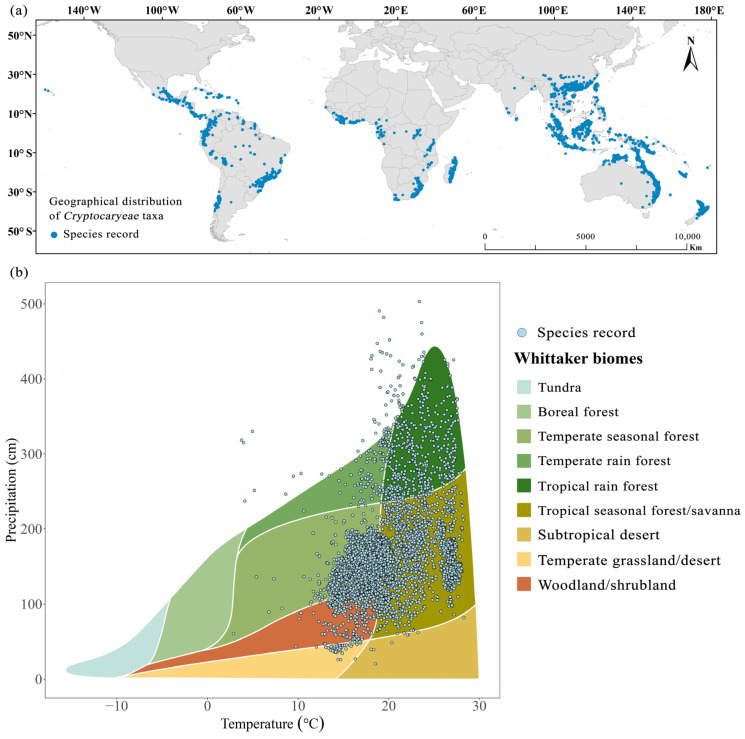
(**a**) The distribution of plant sites included in this study. Each dot may overlap more than one location because of the point size. (**b**) denotes the region of distribution of the biota of the different species in this study.

Whittaker’s biomes analysis suggested that the distribution of Cryptocaryeae populations is concentrated in tropical and temperate rainforests and seasonal forests that are relatively warm and humid, with temperatures and precipitation ranging from approximately 15–28 °C and 100–220 cm. In addition, temperate tropical seasonal forest/savanna biomes have a relatively high species richness in the Whittaker biome-wide grid cells (Figure 4b).

### 2.2. Longitudinal and Latitudinal Gradients of Leaf and Fruit Morphological Traits of Cryptocaryeae Trees

**Figure 1:**
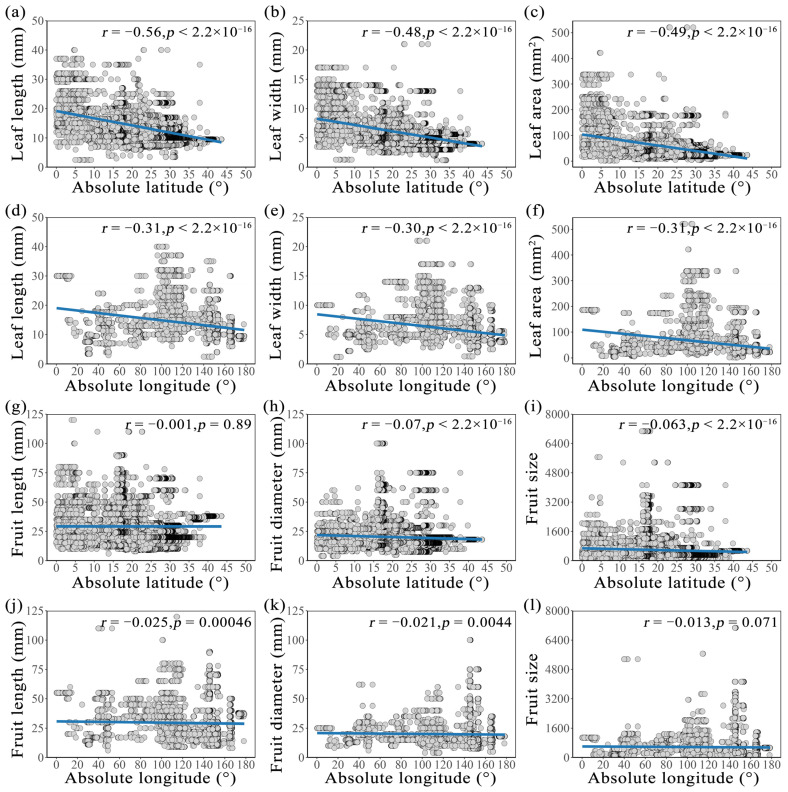
Trends in spatial distribution of leaf and fruit traits of Cryptocaryeae trees. (**a**–**f**) indicate the effects of latitude and longitude on morphological traits such as leaf length, width, and leaf area, while (**g**–**l**) indicate the effects of latitude and longitude on morphological traits such as fruit length, diameter, and fruit size.

For the morphological traits of leaves and fruits in the n tropical Cryptocaryeae trees, the average values for leaf length, leaf width, leaf area, fruit length, fruit diameter, and fruit size are 13.15 mm, 5.64 mm, 51.03 mm^2^, 29.192 mm, 19.72 mm, and 557.40 mm^2^, respectively. The range of leaf length is 2.5–40 mm, the range of leaf width is 1.2–21 mm, the range of leaf area is 2.015–520.8 mm^2^, the range of fruit length is 6–120 mm, the range of fruit diameter is 4–100 mm, and the range of fruit size is 28.27–7068.6. Analysis using a general linear model revealed that leaf and fruit morphological traits in Cryptocaryeae species exhibit relationships with latitude and longitude. We further observed that the magnitude of response differed between the leaf and fruit morphological traits, the leaf morphological traits were influenced by spatial variation along latitudinal and longitudinal gradients (Figure 1). Leaf traits (including leaf length, leaf width, and leaf area) showed a significant negative correlation with latitude (Figure 1a–c, *p* < 0.001, r = −0.56, −0.48 and −0.49, respectively). This indicates that leaves at higher latitudes were significantly shorter, narrower, and smaller in area compared to those in tropical regions. Additionally, a significant negative trend was also observed for leaf traits along the longitudinal gradient (Figure 1d–f, *p* < 0.001, r = −0.31, −0.30 and −0.31, respectively), demonstrating pronounced spatial variability in leaf morphology. In contrast, fruit traits (fruit length, fruit diameter, and fruit size) exhibited no detectable pattern across both latitudinal and longitudinal gradients (Figure 1g–l; r = −0.001, −0.07, −0.063, −0.025, −0.021 and −0.013, respectively).

### 2.3. Climatic Influences on the Leaf and Fruit Morphological Traits of Cryptocaryeae Trees

**Figure 2:**
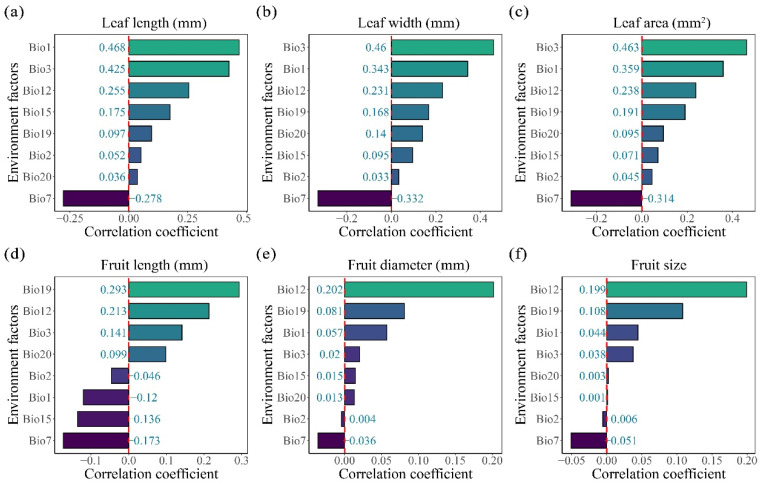
The relationship between leaf and fruit traits of Cryptocaryeae trees and environmental factors. (**a**–**c**) represent the correlation between leaf length, width, and leaf area and environmental factors, respectively. (**d**–**f**) represent the correlation between fruit length, diameter, and fruit size and environmental factors, respectively.

**Figure 3:**
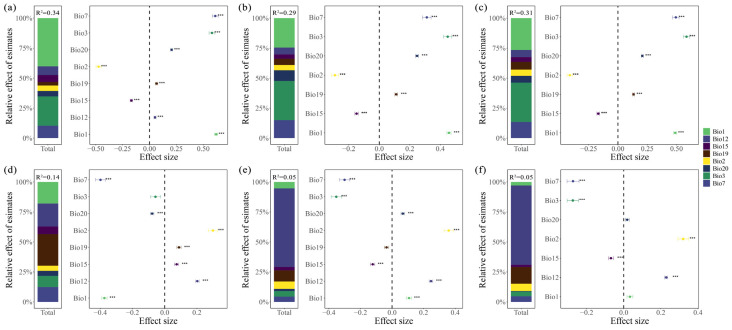
The relative effects of environment factors on leaf and fruit traits of Cryptocaryeae trees. (**a**–**c**) represent the relative importance and contribution rates of environmental factors to leaf length, width, and leaf area, respectively. (**d**–**f**) represent the relative importance and contribution rates of environmental factors to fruit length, diameter, and fruit size, respectively. Here, the significance level is *p* < 0.001.

The relationships between eight environmental factors (Bio1, Bio2, Bio3, Bio7, Bio12, Bio15, Bio19, Bio20) and leaf and fruit morphological traits were analyzed by multiple linear model and correlation analysis. The results showed that leaf traits were significantly influenced by temperature factors such as Bio1, Bio3, and Bio7 (Figure 2a–c and Figure 3a–c). Among these, leaf length, width, and leaf area were all significantly negatively correlated with Bio7 (*p* < 0.001, r = −0.278, −0.332, and −0.314, respectively); Bio3 had the strongest effect on leaf width and leaf area (*p* < 0.001, r = 0.46 and 0.463, respectively), while Bio1 had the strongest effect on leaf length (*p* < 0.001, r = 0.468). Fruit traits were primarily significantly correlated with Bio12 and Bio19 (Figure 2d–f and Figure 3d–f), and fruit length, width, and fruit size were also significantly negatively correlated with Bio7 (*p* < 0.001, r = −0.173, −0.036, and −0.051, respectively). Among these, Bio19 had the strongest effect on fruit length (*p* < 0.001, r = 0.293), while Bio12 had the strongest effect on fruit diameter and fruit size (*p* < 0.001, r = 0.202 and 0.199, respectively).

Through the hierarchical segmentation method to further determine the relative importance of each variable of environmental factors to the variation of leaf size and fruit morphological traits of Cryptocaryeae. The results showed that Bio1, Bio3, and Bio7 had the highest relative importance in the variability of leaf morphological traits in Cryptocaryeae species (Figure 3a–c). The relative contribution rates of these three variables to leaf length were 40.15%, 24.53%, and 10.32% (Figure 3a), and to leaf width were 24.46%, 32.63%, and 14.98% (Figure 3b), and their relative contribution rates to leaf area were 26.57%, 32.78%, and 13.46%, respectively (Figure 3c). Among the fruit morphological trait variations, Bio1, Bio7, Bio12, and Bio19 had the highest relative importance (Figure 3d–f), with relative contribution rates to fruit length of 18.04%, 12.45%, 19.23%, and 26.43%, respectively (Figure 3d). For fruit diameter and fruit size, Bio12 had the highest relative contribution rates, reaching 65.56% and 66.87%, respectively (Figure 3e, f).

## 3. Discussions

In this study, we found that the leaf morphological traits of species in Cryptocaryeae decreased with increasing latitude, and large leaves were mainly distributed in areas with lower latitudes and were significantly affected by environmental factors such as annual mean temperature (Bio1), isothermality (Bio3), and annual temperature range (Bio7). In agreement with the previous studies [12,28,29]. Our results suggest a response of leaf morphological traits to temperature and latitudinal gradients. All leaf traits vary with environmental conditions [30]. These leaf morphological changes are not only a survival strategy for plants to adapt to changes in the environment [14,31], but also a manifestation of effective reflection of changes in the habitat [32,33]. For example, a previous study on Chilean *Myrceugenia* (Myrtaceae) reported that while leaf frost resistance exhibited a lag relative to thermal niches, variation in leaf morphological traits was significantly influenced by microenvironments, demonstrating high evolutionary plasticity [34]. And in cold, dark environments, plants typically adopt conservative resource utilization strategies to ensure survival [35]; conversely, in warm, well-lit, and stable conditions in tropical forests, plants tend to adopt more acquisitive growth strategies [30,36,37]. Furthermore, in low-latitude forest environments, species richness is higher and different plants compete more for resources, so the larger the specific leaf area (SLA), the stronger the plant’s acquisition strategy is likely to be [38]. And an increased exposed area of leaves is associated with more light, favoring the plant’s ability to survive in a light-competitive environment [39,40]. In contrast, large leaves have a significant disadvantage in low-temperature environments at high latitudes, where they are more susceptible to frost damage [29]. Studies have demonstrated that its variation is frequently related to the plant’s response to environmental factors such as light, water, temperature, etc. [41]. Leaves of plants expand as temperatures increase, while leaf expansion is restricted at low temperatures [42,43]. Plants adapted to warm environments often have larger, thinner leaves with lower water use efficiency, reflecting more acquisition strategies, while plants that grow in cold environments are the opposite [36,44].

In contrast, there was no clear spatial pattern of fruit morphological traits at latitude, while fruit functional traits were significantly influenced mainly by precipitation. In tropical forests, approximately 90% of woody plants rely on birds and mammals for seed dispersal [45]. Traits such as fruit size, diameter, color, and taste, are more affected by factors such as the selection of dispersers (e.g., animals), etc. [46], which usually contribute to seed conservation and dispersal [47], and their size and effectiveness of dispersal directly affect the reproduction of populations. For instance, the fleshy fruits of plants are a major food source for many animals [48,49]. In exchange, animals contribute to the reproduction of plant populations by dispersing seeds from the fruit [50]. Moreover, these fruit traits are often interrelated, forming what is termed a “dispersal syndrome” to attract specific dispersers [51]. For instance, fruits relying on birds for dispersal are typically smaller and darker in color, while those dependent on mammals tend to be larger and lighter in hue [45,51,52]. This demonstrates a coevolutionary relationship between fruit traits and frugivores, with variations among frugivores shaping the evolution of fruit characteristics [45]. Therefore, the trait of fruit size undergoes little variability in space and this is closely related to the plant’s survival strategy. Adequate precipitation usually favors plant growth and metabolism, and can provide enough water for fruit development so that fruit cells can absorb enough water to expand, thus promoting fruit enlargement. In contrast, in arid regions, plant fruit traits and propagation strategies may also be affected by the degree of aridity [53], where insufficient water may lead to smaller fruits, and plant propagation strategies may be more focused on quantity [54]. Thus, precipitation has a greater effect on fruit traits.

In the present study, the different responses of leaf and fruit traits spatially and to environmental factors suggest that while different plant organs may interact with each other during environmental adaptation, they may also exhibit different adaptation pathways and mechanisms. Previous studies have pointed out that genetics and environment have a great effect on plant phenotypes [15,55]. Changes in the functional characteristics of plants should enhance their ability to adapt to changes in the local environment [56]. The response to environmental conditions within a same species is both trait-specific and resource-specific, and varies based on genotype [15]. The leaf plasticity is co-regulated by both environment and genetics [57]. However, Studies have shown that the leaf size (leaf length, leaf width, etc.) was not conservative and unstable in evolution [58], and its variation was more affected by continuous environmental selection rather than phylogenetic development [59,60]. For example, The effect of canopy position on leaf size variation during plant growth is also much higher than genotype [61]. While the nutrient organ is not heritable, so the morphological trait of leaf is more affected by the environment, and it is the embodiment of the plasticity that responds to the environmental changes, and the effect of the genetic regulation is smaller. However, fruits are reproductive organs, it was found that plant genome size has a higher effect on fruit seed size than any other phenotypic trait (except lifestyles), suggesting that fruit traits may also be primarily regulated by genetic factors, with environmental and other biotic factors playing a secondary role [62,63]. Additionally, fruit traits, as a reproductive trait, have low phenotypic plasticity [64], and climatic factors explain much less of the variation in seed size, with more significant phylogenetic effects on seed size [65].

In addition, leaves and fruits, as important components of plants, differ somewhat in their functions and ecological niches in the plant [28], and this difference may lead them to exhibit relatively independent environmental adaptations when subjected to different selective factors. For example, and leaf traits are considered to be a major determinant of the trade-off strategy between tree growth and survival, as the larger the specific leaf area (SLA), the stronger the plant’s acquisition strategy [38]. Therefore, the leaf may be more focused on improving photosynthetic efficiency and resource acquisition to adapt to environmental changes [66]. While fruits may be more focused on attracting dispersers and protecting seeds, and as the habits of disseminators (fruit-eating animals) change, they tend to evolve larger and more colorful fruits [67]. The uniqueness exhibited by leaf and fruit size in adapting to the environment is the result of a number of different environmental factors driving the process. Thus, further exploration of the relationships between leaf and fruit functional traits under different plant species and ecological conditions could lead to a more comprehensive understanding of the complexity of plant evolution and ecological adaptation.

Although this study utilizes a global dataset, several limitations should be acknowledged. The sample size is limited in certain regions, and the inclusion of 369 species from a clade of over 850 may introduce phylogenetic and geographic biases. Furthermore, the analysis was conducted primarily at the species level, which constrains our ability to propose mechanistic explanations for the observed patterns. Future research should prioritize genomics as a core approach. It is recommended to integrate methods such as population genomic approaches, such as population genomics, to further elucidate the microscopic molecular mechanisms underlying trait adaptation.

## 4. Materials and Methods

### 4.1. Morphological Trait Data

We obtained occurrence records for accepted Cryptocaryeae taxa from GBIF (https://www.gbif.org), extracting 17,117 data points using R scripts [68]. Specifically, during the initial data collection phase, only records containing geographic reference information were extracted, and those with coordinate values of zero or integers were excluded. Subsequently, data cleaning was performed using an R script. On the one hand, outliers with geographic locations exceeding three standard deviations of the Euclidean distance from the species distribution geographic center were removed; on the other hand, records with lacking environmental data were filtered out [68]. These 17,117 records encompass a total of 369 unique species (Figure 4a). For these species, we collected leaf and fruit morphological trait data, including leaf length, width, and area, and fruit length, width, diameter, and size.

### 4.2. Environmental Factors Data

The environmental data used in this study includes the latitude and longitude information of the distribution points of Cryptocaryeae taxa plants, as well as a total of 20 environmental climate variables [69].

**Figure 5:**
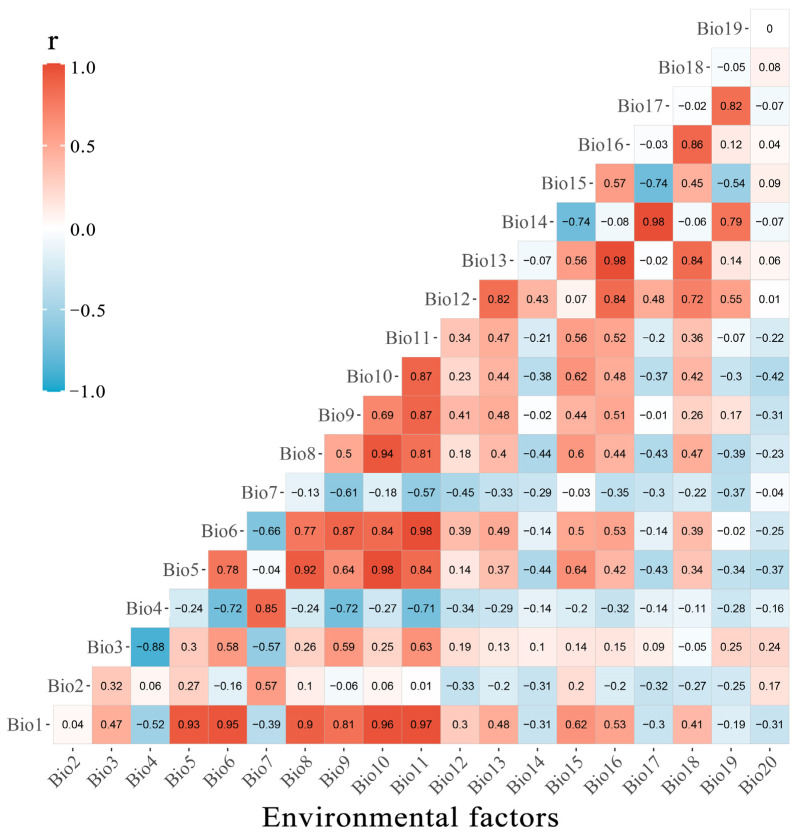
Correlation between 20 environmental factors.

However, in practice, there is often a certain degree of multicollinearity among predictor variables [70]. This issue is difficult to avoid when selecting biologically meaningful variables, as many commonly used environmental predictors are highly correlated or non-independent [71]. Highly correlated variables make it difficult to distinguish their individual independent effects [72]. A practical guideline for handling multicollinearity is to retain variables with correlation coefficients below a specific threshold [70,72]. Accordingly, after excluding variables with Pearson correlation coefficients greater than 0.7 among the 20 environmental climate variables (Figure 5), we ultimately retained eight environmental climate variables for subsequent analysis (Table 1).

### 4.3. Data Analysis

We established a linear model to analyze trends in leaf and fruit morphological trait variation of Cryptocaryeae taxa across latitudes and longitudes. Pearson correlation analysis was performed to determine the collinearity among the 20 environmental factor variables. The “dredge” function in the MuMIn package [73] was used to select the multiple regression model with the minimum AIC (Akaike information criterion) to analyze the effects of environmental factors on the morpho-logical traits of Cryptocaryeae leaves and fruits. And the variance decomposition model was further established by using the hierarchical segmentation method to quantify the relative contribution rate of different environmental factors to the morphological traits of leaves and fruits [74]. At the same time, the correlation between leaf and fruit size of Cryptocaryeae and environmental factors was analyzed to study the mechanism of ecological adaptability. The significance level of all statistical tests was set to 95% confidence interval. All analytical methods and analytical models were completed using R 4.3.3 software [75].

## 5. Conclusions

In this study, we analyzed the relationship between leaf and fruit morphological traits and environment factors of Cryptocaryeae taxa (Lauraceae). We found no consistent spatial variation pattern between leaf and fruit morphological traits, and they were also mainly affected by different dominant environmental factors, indicating that they may be adapted to the environment in different ways. In these tropical and subtropical plants, leaf morphological traits were negatively correlated with latitude and longitude gradient and showed positive sensitivity to temperature, indicating that nutritional traits may adapt to environmental changes mainly through phenotypic plasticity. In contrast, reproductive traits such as fruit morphology showed limited variation along the latitude gradient, which may be mainly affected by survival strategies and genetic control to adapt to the environment. This limited variation may represent a strategy to balance dispersal efficiency and offspring survival within stable tropical niches. Our findings highlight that understanding environmental adaptation in Cryptocaryeae requires integrating ecological drivers (climate mediated plasticity) with genetic evolutionary processes while analyzing multiple functional traits synergistically. In addition, we documented correlational patterns between leaf morphology and climate variables across Cryptocaryeae species, while fruit traits showed no detectable environmental relationships. These patterns warrant experimental investigation to determine their internal mechanisms. Future studies should validate genetic constraints on fruit traits using molecular markers and expand trait networks (e.g., wood anatomy) to model holistic adaptive strategies under climate change.

## Figures and Tables

**Table 1:** Environment factor information.

Code Name	Environmental Factors
Bio1	Annual Mean Temperature
Bio2	Mean Diurnal Range
Bio3	Isothermality
Bio7	Annual Temperature Range
Bio12	Annual Precipitation
Bio15	Precipitation Seasonality
Bio19	Precipitation of Coldest Quarter
Bio20	Digital Elevaton Model

## Data Availability

The data that support the findings of this study are available from the corresponding author, upon reasonable request.
